# A Green Analytical Method Using Ultrasound in Sample Preparation for the Flow Injection Determination of Iron, Manganese, and Zinc in Soluble Solid Samples by Flame Atomic Absorption Spectrometry

**DOI:** 10.1155/2012/298217

**Published:** 2012-03-22

**Authors:** M. Carmen Yebra

**Affiliations:** Department of Analytical Chemistry, Nutrition and Bromatology, Faculty of Chemistry, University of Santiago de Compostela, Santiago de Compostela 15782, Spain

## Abstract

A simple and rapid analytical method was developed for the determination of iron, manganese, and zinc in soluble solid samples. The method is based on continuous ultrasonic water dissolution of the sample (5–30 mg) at room temperature followed by flow injection flame atomic absorption spectrometric determination. A good precision of the whole procedure (1.2–4.6%) and a sample throughput of ca. 25 samples h^–1^ were obtained. The proposed green analytical method has been successfully applied for the determination of iron, manganese, and zinc in soluble solid food samples (soluble cocoa and soluble coffee) and pharmaceutical preparations (multivitamin tablets). The ranges of concentrations found were 21.4–25.61 *μ*g g^−1^ for iron, 5.74–18.30 *μ*g g^−1^ for manganese, and 33.27–57.90 *μ*g g^−1^ for zinc in soluble solid food samples and 3.75–9.90 *μ*g g^−1^ for iron, 0.47–5.05 *μ*g g^−1^ for manganese, and 1.55–15.12 *μ*g g^−1^ for zinc in multivitamin tablets. The accuracy of the proposed method was established by a comparison with the conventional wet acid digestion method using a paired *t*-test, indicating the absence of systematic errors.

## 1. Introduction

 Trace metal ions have important roles in our life because of involving a wide spectrum of activities. Iron, manganese, and zinc are essential trace elements, having several roles in many body functions [1].

Sample preparation is a key procedure in modern chemical analysis because it is one of the most time consuming and error-prone portions of any analytical scheme. In spite of major advances in the instrumentation used in determinative steps, many laboratories use sample preparation techniques that are time consuming and labor intensive. Sample dissolution is one of the most common operations in analytical chemistry. Because most quantitative techniques require that samples are introduced in liquid form, thousands of sample dissolutions are performed every working day in analytical laboratories. However, most conventional digestion procedures involving inorganic acids are tediously labor intensive, and a number of them are hazardous to laboratory workers and to the wider environment [2–4].

In order to avoid these drawbacks, sample introduction as slurry for different techniques can be an interesting approach. However, the use of chemical modifiers is required for slurry analysis by electrothermal atomic absorption spectrometry to obtain interference-free determinations, which increases the analysis cost and can cause sample contamination. However, the main drawback of this technique is the nonhomogeneity of the suspensions formed [5–7]. Ultrasound-assisted sample pretreatments offer an alternative approach to sample preparation prior to analysis. This energy facilitates and accelerates some analytical steps, decreasing the analysis time and the cost per analysis, increasing accuracy and precision (above all in a continuous mode) [8–12]. These methodologies have been applied to water insoluble samples, using a diluted inorganic acid (usually nitric acid) as extraction solution. Nevertheless, only three works were found in the literature reporting the use of continuous ultrasound-assisted approaches for dissolution of water soluble samples. These works were developed by our investigation team and are based on continuous milk powder water reconstitution for determination of zinc and iron, and on calcium and magnesium determination in soluble pharmaceutical tablets [13–15].

The food and pharmaceutical industry needs simple, accurate, and green analytical methods that can be routinely and safely used in the companies' own quality control laboratories. This work was focused on the study of the continuous ultrasound-assisted dissolution of water soluble foods and pharmaceutical samples. The continuous ultrasound dissolution system used is connected to a flow-injection (FI) manifold, which allows the continuous determination of trace metals by flame atomic absorption spectrometry (FAAS). The proposed method is based on the use of nontoxic reagents that makes it friendly both for the operator and the environment.

## 2. Experimental

### 2.1. Apparatus

**Figure 1:**
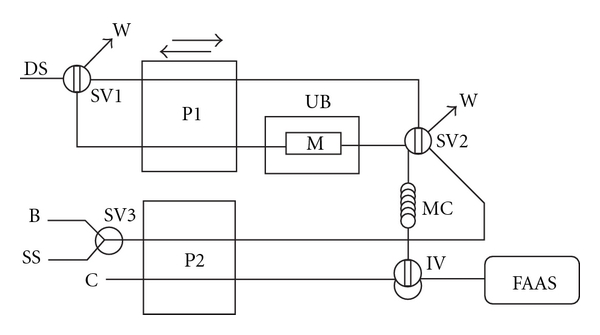
Experimental setup used for the continuous ultrasound-assisted dissolution step and FI determination of iron, manganese, and zinc in soluble samples. P1 and P2, peristaltic pumps; DS, dissolving solution; W, waste; UB, ultrasonic bath; M, minicolumn containing the sample (dissolution cell); SS, standard solution; B, blank; IV, injection valve; SV1–SV3, switching valves; MC, mixing coil; C, carrier (ultrapure water); FAAS, flame atomic absorption spectrometer.

The FI manifold used (Figure 1) has two sections: Section 1 where the solid sample is continuously dissolved, and Section 2 where the dissolved sample is led to the detector (FAAS) where the metals are continuously monitored. This manifold comprises two Gilson Minipuls-3 peristaltic pumps (Gilson, Villiers Le Bel, France) fitted with PVC tubes, an ultrasonic bath with an ultrasound power of 40 KHz (Selecta, Barcelona, Spain), and a glass minicolumn used as sample container (50 mm × 3 mm i.d., bed volume 350 *μ*L) (Omnifit, Cambridge, UK). The ends of the minicolumn were plugged with filter paper (Whatman 541). Four Rheodyne injection or switching valves models 5041 and 5301 (Rohnert Park, USA). A Perkin Elmer Model 5000 atomic absorption spectrometer (Perkin Elmer, Shelton, CT-USA) with a deuterium lamp as background correction system was used for iron, manganese, and zinc measurements. Hollow cathode lamps (Perkin Elmer) operating at recommended current were used. The instrument was set at 248.3, 279.5, and 213.9 nm for Fe, Mn, and Zn determination, respectively. The spectrometer output was connected to a Perkin Elmer Model 50 Servograph Recorder with a range of 5 mV. The signals measured were the heights of the absorbance peaks. Numerical analyses of experimental designs were performed by means of the statistical package Statgraphics Plus 5.1 (Manugistic, Inc. Rockville, MD, USA). 

### 2.2. Reagents and Solutions

All chemicals were of analytical-reagent grade. Iron, manganese, and zinc standard solutions, 1000 *μ*g mL^−1^, were supplied by Merck (Darmstad, Germany). Nitric acid 65% and hydrochloric acid 30% were obtained from Merck (Darmstad, Germany). All glassware used were cleaned in aqueous 4 mol L^−1^ nitric acid for four days and rinsed with ultrapure water before use. Ultrapure water with a resistivity of 18.2 MΩ cm^−1^ was obtained from a Milli-Q purification system (Millipore Co., Bedford, MA, USA).

### 2.3. Procedure

Soluble solid samples were purchased from local markets and pharmacies. Pharmaceutical samples were grinded to a fine powder in a mortar, blended and homogenized, and finally sieved through a 0.1 mm pore diameter plastic sieve. All samples were kept in clean dry containers.

Solid soluble samples (5–30 mg) were directly weighed into the glass minicolumn (dissolution cell). Then, each minicolumn was assembled to the continuous ultrasound-assisted dissolution system, and immersed into the ultrasonic bath. After, the dissolution circuit was loaded with the dissolving solution (1 mL of ultrapure water). The dissolving solution is then circulated at 6 mL min^−1^ through the dissolution cell under ultrasonic irradiation during 2 min at room temperature. The direction of the flow was changed each 30 s to facilitate sample dissolution avoiding sample accumulation in the minicolumn ends. Once the sample is dissolved, the SV2 is switched to its other position, and the sample is led to the mixing coil where the solution is homogenized. Then, the loop of the injection valve (IV) is filled with a volume of 250 *μ*L of the sample solution, which is injected into an ultrapure water carrier stream that transport it at 3.5 mL min^−1^ to the detector.

## 3. Results and Discussion

### 3.1. Optimization of the Continuous Dissolution Step

Six variables, namely, nitric acid concentration, hydrochloric acid concentration, temperature of the ultrasonic water-bath (dissolution temperature), ultrasound exposure time, dissolving solution volume, and flow-rate of the continuous dissolution manifold were selected to be examined. The studied variables as well as the values for each level (low and high) are shown in Table 1.

The consequence of changing a variable from low to high level value was examined on a selected response such as percentage dissolution efficiency, according to the following equation:

(1) $$\%\;\text{dissolution}\;\text{efficiency}\;=\;\left(\frac{A}{B}\right)\;\times \;100,$$

where *A* is the concentration of the metal obtained with the proposed procedure and *B* the metal concentration obtained by a manual dissolution using a conventional digestion procedure with concentrated nitric acid and off-line FAAS detection [16].

A Plackett-Burman 2^*∧*^6*3/16 factorial design with a center point (13 nonrandomized runs) was used to find the main factors affecting the continuous dissolution step. This factorial design was applied to a soluble cocoa sample and to a pharmaceutical preparation (soluble multivitamin tablet). The conclusions of the screening studies reflected that only the ultrasound exposure time was a statistically influential factor, and it was affected by a positive sign. The other variables were not statistically influential factors in the ranges studied at the 95% confidence level and all of them were affected by a positive sign. Although the concentration of the acids used as dissolving solution (nitric and hydrochloric acids) had a positive influence on the dissolution efficiency, a run of the experimental design achieved a quantitative % dissolution efficiency with the minimum value of these variables (0 mol L^−1^). Thus, this minimum value was selected for subsequent experiments for the both factors, and ultrapure water was selected as dissolving solution. The dissolving solution volume and the ultrasonic water-bath temperature had a positive estimated effect, but these parameters yield small values for the main effects. Aiming to increase the analytical sensitivity and to make easy and rapid the sample dissolution step, was chosen as optimum the minimum value of these variables (1 mL and room temperature, 20°C). Likewise, as the flow rate of the continuous ultrasound dissolution system had a positive sign, the maximum value tested for this variable (6 mL min^−1^) was chosen for subsequent experiments. According to the results, a new experimental design shifted in the direction of higher ultrasound exposure times would be desirable. However, this variable was fine tuning outside the framework of the design because a minor number of experiments were required. Then, several experiments were carried out in order to optimize the ultrasound exposure time, resulting in that the optimum value was 2 min for the two kinds of samples studied. All the optimum operational conditions are shown in Table 1.

The sample particle size was studied by a univariate method. For this were tested particle sizes between 0.03–0.1 mm. The results obtained indicated that this variable does not affect to the dissolution process within the range studied.

Other flow parameters involving iron, manganese, and zinc determination were also optimized. Thus, it was verified that with a mixing coil length of 199 cm (equivalent to 1.0 mL, which is the volume of the dissolving solution) was obtained a complete homogenization of the sample solution. The carrier flow rate and the injected sample volume were also studied. The carrier flow rate was tested between 3–6 mL min^−1^, and the injected volume of the sample solution between 100–300 *μ*L. The aspiration flow rate of the nebulizer was adjusted to be the same as the flow rate of the carrier solution. Although the highest aspiration flow rate provides the best sensitivity, a high dispersion takes place because at the same time was increased the carrier flow rate. Therefore, a carrier flow rate of 3.5 mL min^−1^ (dispersion equal to 1.1) was chosen as a compromise to obtain the minimum dispersion in the flow system. With regard to the injected sample volume, it was verified that when the volume was increased, the sensitivity was also increased. Therefore, a volume of 250 *μ*L was chosen as optimum, which in addition allows to inject several times the sample solution to verify its homogeneity.

### 3.2. Analytical Figures of Merit

As can be seen in Table 1, calibration curves were obtained by using a linear plot of the peak height as a function of the standard concentrations of each analyte (*n* = 7). In order to evaluate matrix interferences, the slopes obtained from analytical and analyte addition curves were compared. For this, a cocoa soluble sample and a pharmaceutical preparation (soluble multivitamin tablet) were spiked with several concentrations of the analytes, which were added as dissolving solution into the continuous ultrasound dissolution system. The addition calibration graphs were run (*n* = 7) under the optimal chemical and flow conditions for the whole process. These equations and their corresponding calibration graphs have the same slope, demonstrating that the determination of iron, manganese, and zinc is free of matrix interferences. The limits of detection (LOD) and quantification (LOQ) were calculated as the mass of analyte which gives a signal 3*σ* or 10*σ*, for LOD and LOQ, respectively, above the mean blank signal (where *σ* is the standard deviation of the blank signal, *n* = 30). The values obtained for LOD and LOQ are shown in Table 1. The precision of the continuous analytical method was checked using a sample containing 21.42 *μ*g g^−1^ Fe, 5.74 *μ*g g^−1^ Mn, and 57.90 *μ*g g^−1^ Zn (soluble cocoa), and the results expressed as relative standard deviation (*n* = 11) are also shown in Table 1. The sample throughput of the proposed methodology, taking into account the whole process, was ca. 25 samples per hour.

### 3.3. Analysis of Real Samples

The method was applied to the determination of iron, manganese, and zinc in water soluble food samples and pharmaceutical preparations (soluble multivitamin tablets). The results obtained expressed as *μ*g g^−1^ dry weight and their standard deviation (*n* = 3) are shown in Table 2. The results obtained for food samples were compared with those obtained by employing a wet acid digestion method [16] on same samples by using the paired *t*-test. As is reported in Table 2, both groups of results do not give significantly different values, thus the agreement between them is satisfactory at 95% confidence level.

## 4. Conclusions

The use of a continuous ultrasound-assisted dissolution system coupled to an FI manifold using ultrapure water as dissolving solution is confirmed to be an interesting tool for the automatic dissolution of soluble solid samples of different nature. This continuous manifold allows the dissolution of a sample in a time shorter than that required by a batch sample dissolution procedure. Furthermore, this methodology provides accurate results with high precisions, because there are not sample losses as result of its manipulation, and does not present random errors associated to glassware calibration. The proposed method can be considered as environmentally friendly at every step of the analytical procedure. All these characteristics make the method easy to apply to quality control in industry.

## Figures and Tables

**Table 1:** Factor levels in the Plackett-Burman factorial design with their optimum values and analytical features of the method.

Factor	Low	High	Optimum
HNO_3_ concentration (mol L^−1^)	0	3	0
HCl concentration (mol L^−1^)	0	3	0
Ultrasound exposure time (min)	0.5	5	2
Ultrasonic water-bath temperature (°C)	20	70	20
Flow rate of the continuous ultrasound dissolution system (mL min^−1^)	3.5	6	6
Dissolving solution volume (mL)	1	3	1

Analytical features of the method			

	Fe	Mn	Zn

Calibration graph^a^	*A* = 0.03C + 4.8 × 10^−4^	*A* = 0.062C + 2.2 × 10^−4^	*A* = 0.21C + 5.2 × 10^−4^
Correlation coefficient (*r* ^2^)	0.9997	0.9998	0.9998
LOD (*μ*g mL^−1^)	0.02	0.01	0.03
LOQ (*μ*g mL^−1^)	0.06	0.05	0.1
Relative standard deviation (%)	2.4	4.6	1.2

^a^A, absorbance signal; C, concentration expressed as *μ*g mL^−1^.

**Table 2:** Determination of Fe, Mn, and Zn in soluble foods and pharmaceutical samples and paired *t*-test.

Samples	Concentration, mean ± standard deviation (*n* = 3) (*μ*g g^−1^)					
	Fe		Mn		Zn	
Soluble food sample	Official method	Present method	Official method	Present method	Official method	Present method

Soluble cocoa	20.31 ± 1.63	21.42 ± 0.65	5.31 ± 0.55	5.74 ± 0.21	55.41 ± 2.91	57.90 ± 1.00
Soluble coffee 1	43.77 ± 1.75	44.71 ± 0.71	17.14 ± 0.55	18.30 ± 0.34	54.14 ± 1.87	53.18 ± 0.68
Soluble coffee 2	24.16 ± 1.00	25.61 ± 0.65	15.07 ± 0.80	14.11 ± 0.34	32.22 ± 1.45	33.27 ± 0.68

Concentration, mean ± standard deviation (*n* = 3) (mg per tablet)						

Pharmaceutical preparations	Nominal concentration	Present method	Nominal concentration	Present method	Nominal concentration	Present method

Multivitamin tablet 1	10.0	9.90 ± 0.01	5.0	5.05 ± 0.02	15.0	15.12 ± 0.03
Multivitamin tablet 2	3.6	3.75 ± 0.02	0.5	0.47 ± 0.01	3.0	2.84 ± 0.02
Multivitamin tablet 3	5.6	5.61 ± 0.03	1.4	1.35 ± 0.02	1.4	1.55 ± 0.02

Critical value of *t* (*P* = 0.05) = 2.57; experimental value of *t*: 2.20 for Fe, 0.35 for Mn, and 0.92 for Zn.
